# Spermatogonial Stem Cells in Domestic Animals: Current Insights and Future Directions with a Focus on Dogs

**DOI:** 10.3390/vetsci12111047

**Published:** 2025-11-01

**Authors:** Caterina Squillacioti, Nicola Mirabella, Mario Iasevoli, Simona Tafuri, Valeria Iervolino, Alessandra Pelagalli

**Affiliations:** 1 Department of Veterinary Medicine and Animal Production, University of Naples Federico II, Via F. Delpino 1, 80137 Naples, Italy; caterina.squillacioti@unina.it (C.S.); nicola.mirabella@unina.it (N.M.); simona.tafuri@unina.it (S.T.); valeria.iervolino@unina.it (V.I.); 2 Clinica Veterinaria “Genimal”, Via Principe di Piemonte, 186, Pomigliano d’Arco, 80038 Naples, Italy; marioiasevoli88@gmail.com; 3 Department of Advanced Biomedical Sciences, University of Naples Federico II, Via Pansini 5, 80131 Naples, Italy; 4 Institute of Biostructures and Bioimages, National Research Council, Via De Amicis 95, 80131 Naples, Italy

**Keywords:** spermatogonial stem cells (SSCs), canine testis, dog, reproduction, self-renewal, differentiation, cryptorchidism

## Abstract

The growing interest in improving the fertility-rate of livestock species, considering their high economic value, has prompted the development of new methodological approaches using male germline stem cells. Spermatogonial stem cells’ (SSCs) potential to self-renew and differentiate into mature spermatozoa holds promise for their transplantation into testicular tissue and use in new biotechnological methodologies. Moreover, SSCs’ ability to convey genetic information to the next generation is a property that could be exploited for gene targeting. The review provides an update on the main aspects of SSC biology, focusing on the genetic regulators of self-renewal and differentiation processes and different isolation methods. In addition, recent advancement in the cryopreservation of SSCs from domestic animals and their transplantation into recipients’ testes are also discussed. Finally, a section focused on canine SSCs (cSSCs), their biological aspects, and their potential clinical application in the field of reproduction is included. This represents an effective animal model for human reproduction, development, and disease, given that the reproductive anatomy and physiology of canine species and human are similar. We then report on the potential clinical transplantation of SSCs into recipient testicular tissue and suggest future topics to explore for significant advances in fertility preservation.

## Simple Summary

Over the last few decades, researchers have focused on spermatogonial stem cell (SSC) technology for enhancing livestock reproduction. SSCs isolated from the testes possess a unique capacity for self-renewal or differentiation into functional germ cells and have significant potential for transplantation into testicular tissue. Progress in SSC technology could facilitate the production of transgenic animals with high productive and reproductive traits, as well as the conservation of endangered species. The review summarizes current advancements in the biology of SSCs from domestic animals and methodologies for in vitro culture. In addition, canine SCCs (cSSCs) were examined, representing a valid translational model of human reproduction for the analysis of possible factors influencing fertility in genetic studies. Moreover, future perspectives on SSC transplantation in the testes and other potential clinical applications in reproduction are discussed.

## 1. Introduction

Recently, research on fertility-rate improvement has focused on livestock species, in line with their high economic production value. In fact, the application of reproductive biotechnologies (artificial insemination, embryo transfer, etc.) to shorten the generation interval and improve reproductive performances has been crucial in dairy cattle breeding. However, these biotechnologies have limitations, including a relatively modest rate of genetic improvement in livestock. In this context, the rapid development of new methodological approaches using male germline stem cells has led to numerous possibilities in this field. In particular, spermatogonial stem cells (SSCs) are adult stem cells residing at the basal membrane of the testes’ seminiferous tubules that play a role in normal spermatogenesis by producing countless sperm a day through the reproductive lifecycle. SSCs periodically perform self-renewal and divide to maintain a population of undifferentiated cells while developing progenitors for differentiation. This process is tightly modulated by a complex network of paracrine and autocrine signals [1,2,3], predominantly secreted by cellular constituents of the testicular microenvironment—namely spermatogonia and surrounding somatic support cells, including Sertoli, Leydig, and PTM cells—which together form an “open” stem cell niche. Factors including cytokines, adhesion molecules, and oxygen availability in the niche control intracellular signaling cascades, transcription factor expression, and metabolism influencing the spermatogonia. Recently, SSCs have attracted growing interest for their potential to give rise to ES-like cells with lower immunogenicity and an improved safety profile compared to ESCs, which are associated with ethical concerns and limitations in cell sourcing due to their embryonic origin. These features could make them optimal for applications in regenerative medicine and the treatment of diseases (i.e., infertility or degenerative diseases), although studies on the exact molecular mechanisms controlling this derivation process are still preliminary [4].

Since SSCs are very rare in the testes, their characterization in different animals could facilitate their cultivation *in vitro* for regenerative medicine or other research areas. While the prolonged cultivation of SSCs *in vitro* and the possibility of their transplantation into testicular tissue for regenerative purposes have been elucidated in livestock species, research on domestic animals is scarce. It is worth noting that domestic animals, such as canine species, could represent an optimal animal model for experimental and clinical testing that can be translated to human models [5]. Several findings demonstrating the importance of using species other than laboratory animals have been reported recently [6]. Moreover, since the sequencing of the canine genome [7], and emerging evidence showing that canine and human genetic disorders are similar, interest in this species and its biological characteristics has increased.

Recently, the exploration of innovative methods for culturing SSCs, as well as the influence of the testis microenvironment and several other factors on spermatogenesis, has emphasized the potential of these cells for therapeutic applications. In particular, SSCs could be used in therapies for diseases associated with subfertility/infertility or oncology (complementing other advanced assisted reproductive technologies and various genome-editing tools).

This review explores current knowledge on SSCs from domestic animals, considering their potential for clinical applications in reproduction with a focus on characterizing canine SSCs (cSSCs) and the pathophysiologic conditions affecting fertility. The dog is considered a valid model for the study of human diseases (i.e., genetic and aging-related diseases) [8,9,10]. Recently it has been used to study reproductive anatomy and physiology [11]. Moreover, evidence of environmental pollution’s influence on both human and animal fertility indicates that the dog should be studied as a sentinel species [12].

## 2. Spermatogonial Stem Cells (SSCs): Characteristics and Main Roles

Continuous spermatogenesis generates haploid sperm cells through a series of mitotic and meiotic divisions in the testis and is fundamental for male fertility. It is characterized by highly ordered and cyclic progressive phases of germ cells differentiation through the seminiferous tubules of the testis, meticulously controlled by hormones and cell-signaling mechanisms. Spermatogenesis is fundamentally dependent on the differentiation of a group of adult stem cells called SSCs, which, limited in number in relation to the total cellular population [13], reside at the basal membrane of the testis’ seminiferous tubules, surrounded by a characteristic microenvironment (mainly comprising Sertoli cells, Leydig cells, PTM cells, testicular endothelial cells, macrophages, and the ECM) [14,15,16,17]. SSCs perform self-renewal to balance the stem cell pool and differentiation [18,19] during spermatogenesis, and the dysfunction of these processes can lead to Sertoli cell-only syndrome (SCOS) due to SSC exhaustion [20]. Another characteristic of SSCs is the absence of heterochromatin in the nuclei, distinguishing them from differentiating spermatogonia (SPG). Moreover, these cells can be identified through an *in vivo* functional assay by their transplantation into the tubules of a recipient testis, where they develop into mature sperm to produce offspring as defined in a pioneering study by Brinster and Avarbock (1994) [21]. The origin of SSCs has primarily been investigated in mice, leading to different theories. The latest of these, the “Fragmentation model”, demonstrates how undifferentiated spermatogonia continuously interconvert between As spermatogonia, (presenting as a single cell), and short syncytial states via spermatogonia fragmentation (Apr) [22,23]. Characterization of SSCs and their surrounding cells demonstrated that their reciprocal communication regulates important functions of SSCs, including homing, self-renewal, and differentiation.

**Figure 1:**
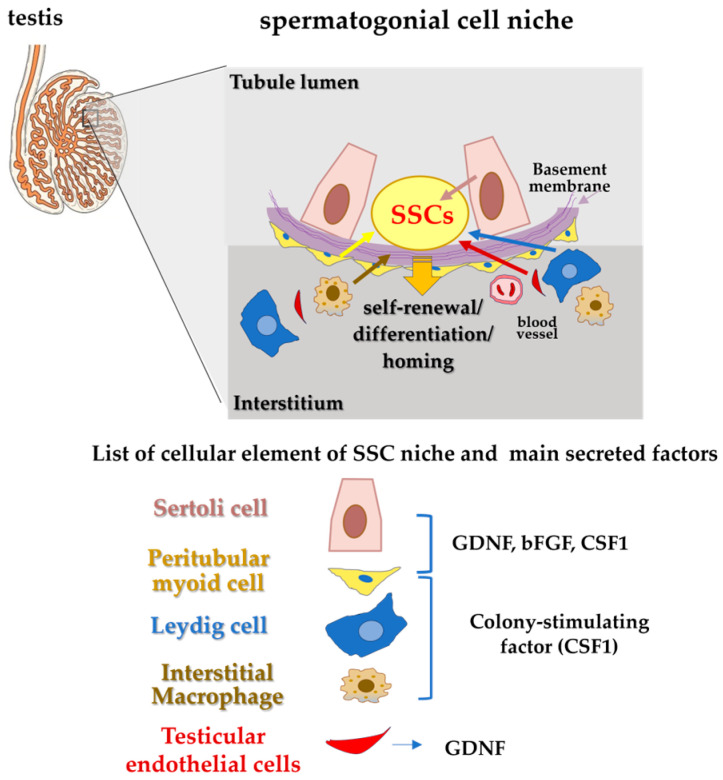
The layout of the SSC niche and a list of main components and secreted factors.

In particular, SSC self-renewal and differentiation are balanced by extrinsic factors from the testicular niche and intrinsic factors within the stem cells themselves. Within the microenvironment of SSCs, Sertoli cells provide physical support and secrete several growth factors and cytokines, including GDNF, FGF2 and CSF1 facilitating the self-renewal and expansion of SSCs (*in vitro* and *in vivo*) (Figure 1) [24,25,26,27,28,29,30].

The main factor responsible for maintaining SSC is GDNF, which binds to GFRα on SSCs and thus enhances downstream signaling pathways (GDNF-RET-GFRα signaling axis). Meanwhile, FGF2, in combination with retinoic acid (RA) promotes spermatogonia differentiation in mouse testicular macrophages [31] (Table 1). Although several studies have investigated GDNF’s activity in different animal models and species, further insights regarding its spatiotemporal regulation are required.

These findings suggest the existence of GDNF- and FGF2-dominant niches [32] and could be used to determine the optimal conditions for SSC expansion *in vitro*. In particular, studies demonstrate that GDNF (40 ng/mL) induces adequate proliferation of ovine SSCs and maintains their stemness for up to 30 days [33]. In addition, the combination of GNDF with basic FGF (bFGF) and GFRα1 is optimal for preserving the stem potential of SSCs and their long-term durability, as shown in a culture of pig SSCs (Table 1) [34]. Other studies have demonstrated that GDNF inhibition leads to the loss of SSCs as well as its related signaling mechanisms, indicating the significant role of this growth factor [35,36]. Other paracrine factors produced by Sertoli cells play important roles in the proliferation of human and mouse SSCs—including IGF1, IGFBP7, NKCC1, and non-receptor type 11 (PTPN11, also known as SHP2) (Table 1) [37].

Another important factor for spermatogenesis is CSF1, which is only expressed in Leydig, Sertoli, peritubular, and spermatogonial cells and has recently been demonstrated in mouse testicular macrophages [30]. Its role in the proliferation and differentiation of spermatogonial cells under *in vitro* culture conditions has been explored in a methylcellulose culture system (MCS) [30].

In addition, several factors other than GDNF (i.e., GATA4, WNT, and NOTCH signaling pathways) could contribute to the growth and self-renewal of SSCs as investigated by *in vitro* studies on mouse bovine, and buffalo SSCs [38,39,40].

**Table 1:** Main factors and gene regulators involved in self-renewal and differentiation of SSCs.

Factor	Role at Level of SSC	Mechanism Involved	Species	Reference
**GDNF**	self-renewal	Nanos2, ETV5, Lhx1, T, BCL6b, Id1, and CXCR4	mouse	[26,35,41]
	self-renewal and proliferation	n.d.	swine	[34]
**IGF1, IGFBP7, NKCC1, and protein-tyrosine phosphatase**	self-renewal and proliferation	CCL24, IGFBP7, and TEK	mouse	[37]
**retinoic acid**	differentiation	downregulation of GDNF expression activation of differentiation factors (BMP and SCF), SOHLH1, SOHL2	mouse, rat	[42,43,44,45]
**PLZF transcription factor**	self-renewal	SALL4 protein	mouse	[46]
**FOXO1 transcription factor**	self-renewal	PI3K-Akt signaling	mouse	[47]
**miR-30**	self-renewal and proliferation	n.d.	mouse	[48]
**miR-34c**	differentiation	Inhibition of the function of NANOS2 gene	mouse	[49]
**miR-202**	self-renewal	Influence of regulators such as STRA8 and DMRT6	mouse	[50]
**miR-17-92 and miR-202**	spermatogenesis	Involvement of Bcl2l11, Kit, SOCS3, and Stat3	mouse	[50,51,52]
**miR-486-5p**	differentiation	up regulating the expression of STRA8 and SYCP3	mouse	[53]
**miR-204**	self-renewal and differentiation	SIRT1	goat	[54]
**bta-miR-146b**	inhibit proliferation and promote apoptosis	n.d.	bovine	[55]

**Abbreviations:** BCL6: B-cell lymphoma 6 (BCL6)-associated X protein; BMP: Bone Morphogenetic Protein; CCL24: C-C motif chemokine ligand 24; CXCR4: C-X-C motif chemokine receptor 4; DMRT6: Doublesex and Mab-3-Related Transcription Factor 6; ETV5: ETS variant transcription factor 5; FOX: forkhead box; Id1: DNA-binding protein inhibitor ID-1; IGF1: Insulin-like growth factor 1; IGFBP: Insulin growth factor-binding protein; Lhx1: LIM homeobox 1; NKCC1: Na^+^-K^+^-Cl transporter isoform 1; PI3K: Akt phosphatidylinositol 3-kinase and protein kinase B; PLZF: promyelocytic leukemia zinc finger; SALL4: Spalt-Like Transcription Factor 4; SCF: stem cell factor; SIRT1: sirtuin 1; SOCS3: suppressor of cytokine signaling 3; SOHLH1: spermatogenesis- and oogenesis-specific basic helix–loop–helix 1; STRA8: stimulated by retinoic acid 8; SYCP3: synaptonemal complex protein 3; TEK: receptor tyrosine kinase.

However, studies on SSC characterization have also analyzed SSC stemness markers, which are similar to those that are found on the surface of other stem cells from different tissues (bone marrow, adipose tissue, etc.). It is worth noting that CD9, which is commonly expressed on other stem cells, has been discovered on the surface of rabbit and mouse SSCs, suggesting its potential association with integrins, including b1- and a6-integrin [56]. Furthermore, several other markers (GPR125, GFR1, THY1, ZBTB16, SSEA-4, and PLZF) have been identified on the surface of rodent and human SSCs, some of which (THY1) have also been found on somatic cells [57,58,59,60,61]. In particular, THY1, a glycosylphosphatidylinositol-anchored glycoprotein of the Ig superfamily, is positively expressed in mouse SSCs. The THY1 gene codes for the thymocyte antigen, which can be used as a marker for a variety of stem cells including SSCs [62,63].

Recently, it was found that CD2 expression in rat and mouse SSCs [64] may be conserved in the SSCs of other animal species. In addition, a new marker named forkhead box protein C2 (FOXC2), with a role in the maintaining the quiescent state of primitive SSCs, has also been identified in an SSC subpopulation in adult mice and humans [65].

However, stemness markers have been left undiscovered for several reasons (low surface antigen recognition, the inefficiency of spermatogonial transplantation, and a lack of long-term culture systems), which has furthered the development of other methodologies requiring SSC enrichment.

Moreover, Illumina high-throughput sequencing technology has improved these studies, proving highly efficient and sensitive for the detection of miRNA expression. Specifically, several miRNAs including miR-21, miR-221, miR-34b/c and miR-449, are important for mammalian spermatogenesis [66]. In-depth studies have demonstrated the expression of these miRNAs in goat CD49f-positive testicular cells and supported by bioinformatic analysis, clarified their role in cell-cycle biological processes [66]. Other studies have shown that RNA transport and MAPK and p53 pathways play vital roles in early SSC differentiation, thus shedding light on the importance of these regulatory mechanisms as possible causes of male infertility [67]. In this regard, it has been demonstrated that miRNA expression is altered by several conditions (i.e., heat stress, xenobiotics) that commonly impact fertility [68]. In addition, a recent study by Qingqing Geng (2025) demonstrated the impact of low levels of vitamin B6 on the miR-1458-TBX6 regulatory axis for SSC formation in Rugao Yellow Chicken [69].

When characterizing “stemness” markers for individual SSCs within cultures of primary testicular cells, the focus is on the specific behaviors of stem cells. In this regard, the increase in colony numbers can indicate the presence of proliferative SSCs [70]. However, there is currently unambiguously established SSC marker for human culture [71].

## 3. Spermatogonial Stem Cells from Domestic Animal Species: Isolation and *In Vitro* Expansion Techniques

Recently, protocols for isolating and enriching SSCs from the testicular tissues of several domestic animal species have been explored. However, following failed SSC isolation attempts (i.e., bovine, swine) [72] using methods employed for rodent species, several critical improvements have been implemented for more consistent success in these procedures [72]. In this regard, a fundamental prerequisite is improving isolation techniques to facilitate the separation and enrichment of this rare population of cells from a larger group of testicular cells [73]. The most widely adopted protocol for isolating SSCs is the enzymatic digestion of testicular tissue (the two–three step protocol) (Table 2) collected from domestic animals at a specific developmental age in order to obtain the greatest cellular population [74].

For ovine species, laminin in combination with bovine serum albumin (BSA) can be used as an appropriate method for SSC isolation from prepubertal ram testes [75] (Table 2). Similarly, for goats in the prepubertal stage, the population of cells that can be isolated from a testis contains a high number of undifferentiated spermatogonia and a few gonocytes, thus exhibiting specific biochemical characteristics related to SSCs [76].

Moreover, the choice of an appropriate medium with the right growth factors for the cell culture, as well as appropriate methods for potentiating SSC renewal and differentiation, is important for prolonging the *in vitro* SSC culture (no longer than 2 months for the majority of animal species) (Table 2) [77,78,79,80].

The role of Sertoli cells as feeder layers in SSC co-culture systems has been well documented. Their supportive effect is reflected in the formation of numerous SSC colonies, as reported in ovine models [81]. This trophic support is closely linked to the secretion of crucial niche components, including GDNF and laminin, which are essential regulators of SSC self-renewal and maintenance [82].

**Table 2:** Protocols for the isolation and enrichment of SSCs from domestic animals.

Animal	Optimal (Time) for Testis Collection (Days)	Isolation (IM) and Enrichment Method (EM)	Factors Added to the Culture Medium	Evaluation Time of SSC Proliferation (Days)	Reference
cat		IM = two-step enzymatic digestion EM = gelatin-coated method	GDNF	43 days	[80]
dog	90–150 (pre-pubertal stage)	IM = CLS digestion	GDNF, FGF2, EGF, soluble GRFA1, LIF, and a laminin substratum	Note: the enriched cells can survive for several weeks	[77]
buffalo	n.d.	IM = two-step enzymatic digestion	FBS (2.5%), GDNF		[83]
calf	150–210	IM = three-step enzymatic digestion 1° (CLS IV), 2° (CLS IV+ HYAL), 3° (trypsin and DNase I) EM = poly-L-lysine-coated method	KSR (15%)	>60	[79]
chicken	21	IM = two-step enzymatic digestion EM = differential plating	FBS (2%), GDNF, bFGF or LIF	7	[84]
goat	120	IM = two-step enzymatic digestion EM = Percoll gradient (32%)	LIF, EGF, bFGF, GDNF	15	[85]
horse	n.d.	IM = two-step enzymatic digestion EM = Percoll gradient (40%)	FBS (10%)	Note: isolated SSCs thawed after cryopreservation demonstrated as much metabolic activity as the fresh cells	[86]
pig	30	IS = two-step enzymatic digestion EM = differential plating (laminin and PLL) in gelatin-coated plates	GDNF, FGF2, IGF1, LIF, EGF	25	[87]
	7–15	IM = two-step enzymatic treatment (CLS, HYAL II, DNase I and trypsin-EDTA) EM = SSC plating in presence of Sertoli cell feeder layer	FGF, GDNF, KSR	>30	[78]
rabbit	90–120	IM = CLS digestion EM = Percoll gradient (32%)	GDNF, FGF2, GRFA1	15	[88]
sheep	n.d.	IM = two-step enzymatic digestion EM = Ficoll gradient (12%) and plating (laminin 20 μg/mL in combination with BSA)	GDNF, EGF, IGF1	30	[33]

**Abbreviations**: BSA: bovine serum albumin; CLS: collagenase; EGF: epidermal growth factor; FBS: fetal bovine serum; FGF: fibroblast growth factor; GDNF: glial cell-derived neurotrophic factor; GRFA1: glial cell line-derived neurotrophic factor family receptor alpha 1; HYAL: hyaluronidase; IGF: Insulin-like growth factor; KSR: knockout serum replacement; LIF: leukemia inhibitory factor.

It has been widely demonstrated that GDNF is a principal growth factor that can promote the *in vitro* proliferation of porcine SSCs [87], suggesting its potential role across different species [83,85] (Table 2). Additional factors, including FGF2, IGF1, and LIF, facilitate porcine SSC proliferation, guaranteeing their survival for more than 25 days with a particular cellular morphology and the formation of grape-like colonies (Table 2) [87].

In addition, experiments on neonatal and adult Swiss albino mice demonstrated a more prolonged culture for SSCs that were isolated from young testes compared with that in adult testes, with a concomitant expression of pluripotency markers (GRFA1, CD9, Nanog, Oct4, and Sox2) [89]. A similar behavior was observed for bovine SSCs [90], suggesting that the animal’s age, and thus the testis development status, is crucial for the success of an SSC culture system. Recently, a hypothesis was developed by Xiao-Yuan Zhang et al., who demonstrated differences in the transcript profiles of prepubertal buffalo (PUB) and adult buffalo (ADU) seminiferous tubules [91]. The enrichment of genes related to SSCs development in PUB compared with ADU suggests that germ cells grow at this age, alongside the morphological development of the testes. This was furthered by the seminiferous tubule’s simple structure in PUB, where the stage of the SSC niche is more established than that in ADU [91].

SSC characterization in other animal species including chickens and cats has been minimally investigated [80,84]. An interesting study focused on establishing suitable conditions for the cryopreservation and storage of spermatogonial stem cells (SSCs) for equine species [86] in which SSC renewal activity was observed after cryopreserved SSCs, were thawed, demonstrating the efficiency of SSC cryopreservation [86] (Table 2). Other studies investigated the optimal culture medium for bovine SSCs to improve their *in vitro* cultivation as well as the epigenetic mechanisms of their proliferation [92]. For example, an interesting paper by Huan Cai et al. demonstrated the efficacy of 2i medium in improving SSCs proliferation and concomitantly reducing the risk of differentiation [92]. Similarly, Rui Yang et al. showed the relationship between H3K9me3 levels and bovine SSCs proliferation [93]. All these findings indicate new reference conditions for bovine SSCs *in vitro* expansion thus improving germplasm cryopreservation and optimizing the culture system for livestock germline stem cells.

However, other factors, including hormones mediating the interaction between germ cells and Sertoli cells during spermatogenesis, are involved in the survival of germ cells [94]. Notably, it has been demonstrated that equine CG (eCG), known to have similar activity to FSH, can influence SSC proliferation by increasing the proportion of colonies relative to the control SSCs [95] (Table 3). In addition, studies on prolonging SSCs’ survival in culture have shown the efficacy of melatonin supplementation (100 μM) in improving cell viability and colony formation, suggesting its pivotal role for mouse SSC development *in vitro* [96] (Table 3).

The hormone added to the freezing medium can also protect frozen–thawed goat SSCs from cellular damage by activating their antioxidant defense system and mitigating excessive freeze-induced autophagy impairment [97]. A similar mechanism involving its interaction with the MT1 and MT2 receptors expressed in several germ lines in seminiferous tubules has been demonstrated to be responsible for SSCs proliferation in rams [101].

More recently, low concentrations of testosterone (60 μg/mL) have been shown to significantly improve the colonization and viability of goat SSCs in a coculture with Sertoli cells, suggesting this hormone’s role in improving SSC culture conditions and thus achieving future progress in reproductive technologies (Table 3) [98].

A study by A. Jafarnejad et al. [99] similarly demonstrated the possible beneficial use of antioxidants (vitamin C and α-tocopherol analog) to counteract oxidative stress and apoptosis, the most common injuries in SSCs [102]. Both products, used separately at an optimal dosage (50 µg/mL of vitamin C or 25 µg/mL of Trolox), improved the viability and colony formation of bovine SSCs after seven days of culturing and reduced apoptosis levels through the regulator BAX and anti-apoptotic BCL2 (Table 3) [99]. Similar beneficial effects on SSC viability have also been demonstrated for ovine species (Table 3) [102].

Although research on SSC culture protocols highlights key factors for improving prolonged culturing of these cells, it is important to clearly state that, to date, the only fully established and reproducible *in vitro* culture and transplantation protocols that exist target murine SSCs [40]. The protocols described herein for other animal species remain limited and largely experimental, facing key challenges in identifying specific markers, achieving long-term proliferation, and enabling efficient colonization after transplantation. In summary, from the literature analysis, it is possible to track a gradient of technological development to guide researchers working with SSCs. Taking rodent SSCs as a well-developed example, well-established conditions allow for the long-term cultivation, self-renewal, genetic manipulation, and recognition of specific factors for cell identification. On the other hand, livestock SSCs represent a developing field of research requiring further improvement (i.e., discovery of validated protocols for the long-term preservation of SSCs from both mature and adult livestock). Canine/feline SSCs currently represent the least developed technology, documenting several limitations for their cultivation (short term-culture and difficulties in SSCs isolation due to the co-existence of other cells).

## 4. Spermatogonial Stem Cells from Domestic Animal Species: Current Insights into Cryopreservation and Transplantation Techniques

As previously reported, the overall goal is to use SSCs for transplantation. The development of this technology is important to evaluate novel insights regarding the entire spermatogenesis process and improve germline repopulation [103,104]. Several aspects need to be considered to guarantee successful transplantation, including the following: a. knowledge of the spermatogenic process; b. the possibility of cryopreserving SSCs; c. methods of producing sterile host males; d. the enrichment of donor SSCs.

Regarding cryopreservation methods for testicular tissue or SSCs, several papers published in 2025 [105,106,107,108,109,110] describe advancement in common methods including soft freezing and vitrification often demonstrating that the latter could be a promising technique (e.g., for calf tissue) [110]. Furthermore, DMSO was suggested as the optimal cryoprotectant preserving both cellular integrity and molecular functionality [111,112]. Furthermore, the efficacy of DMSO-based protocols has been demonstrated for equine species showing that SSCs retain strong viability and expression of stemness markers after cryopreservation [86]. In addition, adding a high level of serum (FBS, 80–90%) to DMSO for sheep SSC cryopreservation, improved post-thaw viability and stemness was observed [107].

However, the comparison of cryopreservation methods for several animal species suggests that further consideration of the specific characteristics and complex regulatory mechanisms of testicular tissue is necessary to optimize protocols in the future. These processes could circumvent several limitations for endangered and rare wildlife (e.g., the scarce long-term conservation of spermatozoa and limited opportunities to collect semen from juvenile animals) preserving genetic diversity and thus potentially reducing the risk of extinction [106]. Moreover, regarding SSCs cryopreservation and transplantation, a clear trajectory of technological development can be observed across species with rodents representing the most advanced models, large species at an intermediate stage of refinement, and canine/feline research still in its early exploratory phase.

The cryopreservation of SSCs is often the step prior to their transplantation in the allogenic or xenogenic recipient. Specifically, the SSC donor and recipient are of the same species in allogenic transplantation, while xenogeneic transplantation uses SSCs from a donor of a related species. Most studies related to transplantation technologies in domestic animal species are shown in Table 4. In most cases, SSC xenotransplantation attempts in domestic animal species failed because incomplete spermatogenesis in the recipient testis due to evolutionary differences among species, whereas most successful results were obtained for rodent species (not mentioned in this table).

Recently, Segunda et al. [118] demonstrated for the first time that SSCs along with allogeneic bovine fetal AT-MSCs, and adult PB-MSCs, can be integrated into the germinal epithelium of seminiferous tubules in recipient bulls’ testes [118]. PB-MSC colonization in the testis was also demonstrated two weeks after transplantation [118].

For transplantation processes to succeed, the endogenous donor SSCs isolated from the testicular tissue and transplanted into the recipient’s testis have to pass through the vas deferens, rete testis, and seminiferous tubules, at which point donor-derived spermatogenesis is then re-established in the microenvironment, namely, the niche [119,120].

Moreover, anatomical differences among the animal species as well as specific technical protocols for SSC injection in transplantation have emerged as important factors [121].

Recently, several research studies have focused on physio-chemical methods (i.e., busulfan, heat/chemotherapy treatment, etc.) to obtain completely sterile recipient testes for more successful SSC transplantation. Many results obtained for different animal species and dosages have displayed adverse effects suggesting the need for new approaches. Recently, the use of fractionated chemotherapy (FC) to obtain sterile, depleted endogenous germ cells in newly hatched chicks was demonstrated to be an optimal strategy, minimizing overall side effects and mortality [114]. In addition, this study demonstrated the first successful xenogenic transplantation of quail SSCs into the testis of a chicken recipient with complete spermatogenesis [114].

However, studies have been dedicated to overcoming the adverse effects of physio-chemical methods. In large animals, a genetic modification such as CRISPR/associated protein 9 (Cas9)-mediated NANOS2 knockout—can genetically sterilize recipients with intact testicular architecture, offering a superior alternative [122,123]. In pigs, DAZL knockout further illustrates the potential of germline ablation, though donor SSC engraftment remains suboptimal [124]. The potential of gene-editing techniques and their ability to sterilize individuals without affecting the genotype transmission of donor offspring has recently been demonstrated in poultry [125] broadening the range of applications for this method, as a way to enhance efficiency and safety in conserving favored poultry species.

## 5. Potential Effects of Xenobiotic and External Factors on the Biology of Spermatogonial Stem Cells

Research first began exploring the possible interference of xenobiotics and substances with spermatogenesis in the 1970s. Since then, specialized research has demonstrated that different conditions altering the testicular microenvironment, as well as the effect of xenobiotics, can impact the biology of SSCs and, thus, their suitability for therapeutic use. In fact, moderate hypoxia (2.5% and 5% O_2_) can improve the proliferation of mouse SSCs, while severe hypoxia induces cells to enter a state of quiescence [126]. Moreover, environmental conditions, such as a high temperature inducing heat stress, can reduce fertility. However, an interesting study on stallion testes under both normal and cryptorchid conditions (used as a model to evaluate the impact of heat stress) demonstrated that undifferentiated SSCs are not more affected by long-term exposure to heat stress than other germ cells involved in spermatogenesis [127]. The authors ascribed this result to the incomplete development of the testis under cryptorchid conditions. On the other hand, using *in vitro*-cultured SSCs, Jia Wang and co-authors [128] illustrated that a high temperature (45 min of 43 °C) can alter the SSCs’ self-renewal ability due to SSC cycle arrest. These data demonstrate the inhibition of the JAK/STAT signaling pathway, commonly known for mediating cell proliferation, differentiation, and migration.

In addition to the effect of environmental conditions on the male reproductive tract and its components, research has also focused on endocrine-disrupting chemicals (EDCs), considering their widespread use [129,130]. EDCs cause a decline in male reproductive health by interfering with the synthesis and mechanism of action of hormones and their detrimental effects have been shown on several function. In this regard, genistein (GEN) and MEHP exposure has been demonstrated to alter the eicosanoid pathway involved in the differentiation of human SSCs [131]. Similarly, bisphenol and two of its main analogs, bisphenol-F (BPF) and bisphenol-(BPS), have been demonstrated to exert cytotoxic effects on SSCs [132,133,134].

Recently, other mechanisms including oxidative stress have been determined as the main cause of apoptosis for porcine SSCs by zearalenone (ZEA) [15–20 μM], a toxic metabolite produced by fungi (such as *Fusarium*, *Aspergillus*, and *Penicillium*) [135].

It is worth noting that chemotherapy has a severe impact on spermatogenesis. Studies in both human and mice have demonstrated that spermatogonia are more susceptible to this treatment than other haploid germ cells [136]. Using an *in vitro* culture of mouse testes, it has recently been demonstrated that cisplatin and doxorubicin exert negative effects on germ cell development by acting directly on SSCs [137]. Similar results have also been demonstrated for the total germ-cell count (including spermatogonial stem cells) in prepubertal human testicular tissue [138].

## 6. Canine Spermatogonial Stem Cells: Characteristics, Pathophysiological Conditions Affecting Fertility and Methods for Their Transplantation

Research on canine spermatogenesis, as well as the characterization of SSCs (cSSCs), has demonstrated that this process develops later in life in this species compared to farm animals and in rodents. In particular, for this species, in-depth information on the isolation methods and *in vitro* culture protocols exists. It is worth noting that spermatogenesis begins at 7 months of age in dogs, and the complete cycle lasts approximately 60 days [139].

Differences in spermatogenesis for other species have been demonstrated, showing that canine type A spermatogonia are stem cells (SSCs or As). These cells are able to self-renew and proliferate, as well as to create, by means of mitosis, intermediate cells and type B cells, which divide to generate primary spermatocytes [139,140,141].

**Figure 2:**
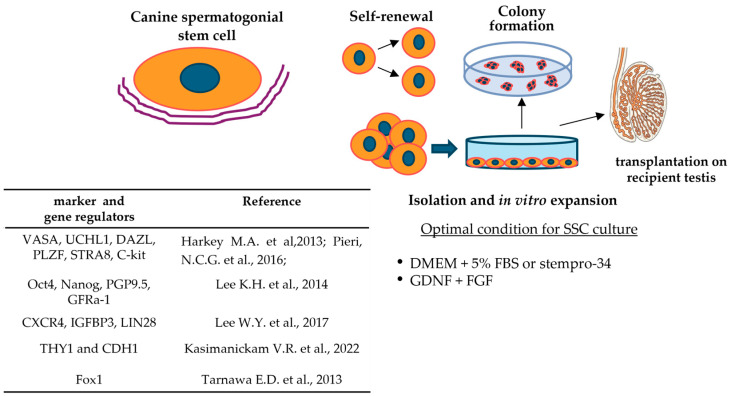
Main characteristics of canine spermatogonial stem cells [77,141,142,143,148,150].

Since they were first identified in 2013 [77], cSSCs have been investigated for specific individual markers, as well as genes regulating self-renewal and differentiation processes (Figure 2). These studies have been conducted either in canine testes (at different stages of the spermatogenesis cycle) or *in vitro*-cultured cSSCs, demonstrating higher expression levels under the latter condition [142].

The studies demonstrated the expression of several markers, including the CXCR4, IGFBP3, LIN28, and SALL4 genes, in different developmental stages of canine testes [143], although significantly different immunohistochemical distributions of IGFBP3 and LIN28, were observed, which exhibited higher expression levels along SCP3-positive differentiated male germ cells.

Recently, characterization studies of cSSCs cultured *in vitro* and in the presence of FSH confirmed the presence of the early germline marker OCT4 and demonstrated the expression of the late germline markers PLZF, DAZL, C-kit, and GFRA-1 [144], thus indicating a morphological profile similar to mouse SSC cultures [145]. In particular, C-kit, a marker of spermatogonial differentiation which is also responsible for this process when spermatogonia self-renewal is repressed, was detected at a higher percentage (33.3%) than in humans (only 13% of cells) [141]. In addition, FSH supplementation influenced the self-renewal of these cells, as well as their proliferation, by activating the GDNF-GFRα1 signaling pathway [144]. This study on canine species is of particular interest, considering that similar research has not yet been carried out for other animal species (rat, mouse, human) [145,146,147].

Recently, it has been demonstrated that the mRNAs of THY1 and CDH1 (which are cell-specific to spermatogonia) are expressed in greater abundance in immature canine testes [148]. The importance of THY1, a surface marker of undifferentiated spermatogonia, was recently evidenced in bull testes, demonstrating that THY1+ cells are enriched in the total testis cell population [149].

Among the regulators of SSC maintenance, Fox1 plays an important role in spermatogenesis, considering its specific expression in uSPG and that its inactivation leads to severe defects in SSC maintenance and differentiation [150] (Figure 2).

SSCs represent a critical component of male fertility, ensuring the lifelong production of spermatozoa through tightly regulated processes of self-renewal and differentiation. These processes are highly sensitive to various pathophysiological conditions, which may disrupt the SSC niche and impair spermatogenesis. One of the most common disorders affecting canine testis is cryptorchidism, a testicular congenital failure that leads to increased intra-abdominal temperature and oxidative stress, resulting in germ-cell apoptosis and a reduced expression of key SSC markers such as PGP9.5, VASA, and DAZL [151,152,153]. The mechanisms behind spermatogenic failure in cryptorchidism have not been fully elucidated. Elevated intratesticular temperature induces oxidative stress, triggering germ-cell apoptosis and impairing spermatogenesis [154]. Experimental models of unilateral cryptorchidism in mice have confirmed early DNA fragmentation and progressive germ-cell loss, which are more severe in adults than juveniles [155]. Species differences also emerge, with rodents showing disrupted spermatogenic differentiation [156].

Chronic asymptomatic idiopathic orchitis (CAO) is another relevant condition that has been associated with non-obstructive azoospermia (NOA) and is characterized by the altered localization and expression of SSC-associated proteins, including FOX and DAZL [157]. At the cellular level, species-specific differences in DAZL localization have been reported. In dogs, DAZL has been predominantly observed in the cytoplasm of both undifferentiated and differentiating spermatogonia, as well as spermatocytes, whereas in mice a dynamic transition from nuclear to cytoplasmic localization occurs during meiosis [141].

Although the pathogenesis of CAO is not fully understood, emerging data suggests that subclinical infections may play a contributory role, for example, the fungal pathogens, *Cryptococcus neoformans* and *Cryptococcus gattii*. These fungi, primarily associated with systemic and central nervous system infections, have been sporadically implicated in subclinical testicular infections leading to chronic inflammation [158,159,160]. A recent case report described a rare instance in which a dog presented with two distinct testicular tumors—a Sertoli cell tumor and an interstitial (Leydig) cell tumor—occurring concomitantly in the same testis, along with fungal infection consistent with mycosis (aspergillosis) [161].

Testicular tumors, including Sertoli cell, seminoma, and Leydig cell tumors, are known to disrupt the architecture and function of the seminiferous epithelium, thereby compromising the SSC niche. In such neoplastic contexts, the integrity and function of SSCs can be significantly impacted, either through direct architectural disruption or via alterations in the testicular microenvironment. Fungal infection further suggests a predisposition due to local immunosuppression or tumor-induced tissue susceptibility. The mechanisms by which such infections influence SSC populations remain poorly defined but may involve immune-mediated destruction of the germinal epithelium and changes in the microenvironment that are detrimental to SSC maintenance. Collectively, these findings highlight the vulnerability of the canine SSC pool to diverse pathological insults and underscore the need for further research into strategies that preserve SSC integrity and function in the context of reproductive disease.

Successful transplantation using of canine seminiferous tubule cells was carried out with recipient canine testes that were previously irradiated to deplete their endogenous male germ cells [162]. A different irradiation approach was carried out using Busulfan treatment (15–17.5 mg/kg), given its ability to deplete germ cells and disrupt the junctions between Sertoli cells, thus enabling the migration of transplanted spermatogonia [163]. The results, demonstrating successful germ-cell depletion in the canine recipient testes sustained for at least eight weeks after treatment, suggest that this method could be useful for preparing recipient testes for transplantation. However, considering the toxic effects of busulfan, surgically induced cryptorchidism was proposed as an alternative method [152].

Other experimental studies using cSSCs in mice showed that supplementing a culture medium of cSSCs with FSH improved their colonization along mouse seminiferous tubules *in vivo* after xenotransplantation [144]. This result was supported by the presence of GFP+ cSSCs along the basal layer of the tubules and the increased percentage of seminiferous tubules positive for GFP+ cSSCs 10 weeks (70 days) after transplantation. Other researchers examined the possibility of transplanting vitrified canine testicular cells into nude mice, demonstrating that SSCs can colonize the seminiferous tubules of the recipient, although spermatogenesis was incomplete [164]. The authors concluded that the microenvironment of mouse seminiferous tubules is unsuitable for domestic animal-derived germ-cell transplantation. The lack of robust protocols for the cryopreservation of canine testicular tissue and the respective cellular component has been expressed as a limitation in a recently published review on canine and feline species. The authors, observing significantly improved results in cats with respect to dogs, suggest the need for advanced studies to potentiate technologies for reproduction in dogs and applications in wild canids [165].

## 7. Conclusions

Although murine SSCs and their *in vitro* culturing remain key reference point for other animal species, further investigations must consider technical and methodological difficulties (i.e., inadequate specific antibodies for SSC characterization, culture medium and supplements for *in vitro* culture, single-cell transcriptome profiling, etc.). Particular attention should be paid to the long-term *in vitro* culturing of SSCs and to their applications in both medicine and agriculture, as well as in rare and endangered wildlife conservation. Canine SSCs (cSSCs) are especially promising for both veterinary and translational medicine, although many challenges still need to be addressed. Key future directions include the development of non-toxic sterilization methods for male recipients using targeted molecular or pharmacological approaches; the development of *in vitro* culture systems capable of sustaining long-term cSSC proliferation with a stable phenotype; the use of cSSCs as models for gene editing and disease modeling in companion animals; the application of cSSCs for fertility preservation, particularly in high-value breeding dogs or endangered canid species; and the investigation of extracellular matrix components and niche signaling in the canine testis to support SSC self-renewal and differentiation.

In addition, a key topic still in its development phase concerns organoid and 3D culture technologies, which would provide new perspectives for canine SSC research. For example, these technologies could enhance testicular development and physiology investigations and support the recreation of a functional testicular microenvironment *in vitro* to support long-term culture. In this regard, some studies have been performed in human and farm animal species, demonstrating the increasing interest and clinical potential of these approaches in areas such as fertility preservation strategies and regenerative medicine [166,167].

Addressing these gaps would lead to significant advances in fertility preservation, canine breeding, and biomedical research using dogs as translational models.

## Figures and Tables

**Table 3:** Factors improving biological aspects of SSC isolated from animals and cultured *in vitro*.

Animal	Factor	Influence on Biological Aspects of SSCs	Reference
mouse	melatonin [100 µM]	cell viability improvement	[96]
goat	melatonin [1 μM] added to the freezing medium	cell viability improvement during cryopreservation	[97]
	testosterone [60 μg/mL]	improvement in cell viability and colonization	[98]
calf	eCG [5 IU/mL]	cell-colony formation	[95]
	vitamin C [50 µg/mL]	improvement in cell viability and colonization	[99]
	α-tocopherol analog [5 µg/mL]	improvement in cell viability and colonization	[100]
sheep	melatonin [10^−7^ M]	improvement in cell differentiation	[101]
	vitamin C [50 µg/mL]	cell viability improvement	[102]

**Table 4:** Main SSC transplantation techniques in domestic animals.

SSC Transplantation				
Allogenic		Xenogenic		
Donor and Recipient	Reference	Donor	Recipient	Reference
calf	[113]	quail	chicken	[114]
chicken	[115]	(testicular tissue)		
goat	[113]			
pig	[113,116]	cat	mouse	[106,117]

## Data Availability

No new data were created or analyzed in this study.

## Abbreviations

As	spermatogonia type A
AT-MSCs	adipose-derived mesenchymal stem cells
BAX-BCL2	B-cell lymphoma 2 (BCL2) -associated X protein
BPF	4,4′-dihydroxydiphenylmethane
BMP	Bone Morphogenetic Protein
BPS	4,4′-sulfonyldiphenol
BSA	bovine serum albumin
CDH1	cadherin 1
CCL24	C-C motif chemokine ligand 24
CLS	collagenase
CSF1	and colony stimulating factor 1
CXCR4	C-X-C motif chemokine receptor 4
CRISPR	clustered regularly interspaced short palindromic repeats
DAZL	deleted in azoospermia-like
DMEM	Dulbecco’s Modified Eagle Medium
DMSO	dimethyl sulfoxide
DMRT6	Doublesex and Mab-3-Related Transcription Factor 6
eCG	equine chorionic gonadotropin hormone
ECM	extracellular matrix
EDCs	endocrine-disrupting chemicals
EGF	epidermal growth factor
ESCs	embryonic stem cells
ETV5	ETS variant transcription factor 5
FBS	fetal bovine serum
FGF2	fibroblast growth factor 2
FOX	forkhead box
GATA4	GATA Binding Protein 4
GDNF	glial cell-derived neurotrophic factor
GFP	green fluorescent protein
GRFA1	glial cell line-derived neurotrophic factor family receptor alpha 1
H3K9me3	histone 3 lysine 9 trimethylation
HYAL	hyaluronidase
Id1	DNA-binding protein inhibitor ID-1
IGF1	insulin-like growth factor 1
IGFBP	Insulin growth factor-binding protein
JAK/STAT	Janus Kinase/Signal Transducer and Activator of Transcription
Kit	c-kit proto-oncogene
KSR	knockout serum replacement
Lhx1	LIM homeobox 1
LIF	leukemia inhibitory factor
MAPK	mitogen-activated protein kinase
MCS	methylcellulose culture system
MEHP	mono(2-ethylhexyl) phthalate
MRT6	mab-3-related transcription factor B1
MSC	mesenchymal stromal cells
MT1/MT2	melatonin receptor 1A/melatonin receptor 1B
Nanos2	nanos C2HC-type zinc finger 2
NKCC1	Na^+^-K^+^-Cl transporter isoform 1
OCT4	octamer-binding transcription factor 4
PB-MSCs	peripheral blood-derived mesenchymal stem cells
PGP9.5	protein gene product 9.5
PI3K-Akt	phosphatidylinositol 3-kinase and protein kinase B
PLZF	promyelocytic leukemia zinc finger
PTM	peritubular myoid
PTPN11	protein tyrosine phosphatase and non-receptor type 11
RA	retinoic acid
SALL4	spalt-like transcription factor 4
SCF	stem cell factor
SIRT1	sirtuin 1
SOCS3	suppressor of cytokine signaling 3
SOHLH1	spermatogenesis- and oogenesis-specific basic helix–loop–helix 1
SSCs	spermatogonial stem cells
STAT3	signal transducer and activator of transcription 3
Stra8	stimulated by retinoic acid 8
SYCP3	synaptonemal complex protein 3
TEK	receptor tyrosine kinase
ZEA	zearalenone
